# Social content and emotional valence modulate gaze fixations in dynamic scenes

**DOI:** 10.1038/s41598-018-22127-w

**Published:** 2018-02-28

**Authors:** Marius Rubo, Matthias Gamer

**Affiliations:** Department of Psychology, Julius Maximilian University of Würzburg, Würzburg, Germany

## Abstract

Previous research has shown that low-level visual features (i.e., low-level visual saliency) as well as socially relevant information predict gaze allocation in free viewing conditions. However, these studies mainly used static and highly controlled stimulus material, thus revealing little about the robustness of attentional processes across diverging situations. Secondly, the influence of affective stimulus characteristics on visual exploration patterns remains poorly understood. Participants in the present study freely viewed a set of naturalistic, contextually rich video clips from a variety of settings that were capable of eliciting different moods. Using recordings of eye movements, we quantified to what degree social information, emotional valence and low-level visual features influenced gaze allocation using generalized linear mixed models. We found substantial and similarly large regression weights for low-level saliency and social information, affirming the importance of both predictor classes under ecologically more valid dynamic stimulation conditions. Differences in predictor strength between individuals were large and highly stable across videos. Additionally, low-level saliency was less important for fixation selection in videos containing persons than in videos not containing persons, and less important for videos perceived as negative. We discuss the generalizability of these findings and the feasibility of applying this research paradigm to patient groups.

## Introduction

Like most vertebrates, humans can only obtain a part of their visual field at a high acuity and therefore repeatedly move their eyes in order to construct a representation of their environment with sufficiently high resolution^1^. Controlling gaze along with retrieving and filtering relevant signals from the environment is a central task of the attentional system^2^. In the past, various lines of research have addressed the mechanisms driving such attentional control.

As sociability is one of human’s key features^3^, a large body of research has assessed how we gather social information in order to infer other persons’ intentions and feelings. For instance, it was shown that socially relevant features like human heads and eyes^4,5^, gaze direction of depicted people^6^, people who are talking^7^ and people with high social status^8^ attract attention when freely viewing images or dynamic scenes. However, non-social cues like text^9,10^ and the center of the screen^11–13^ can also serve as predictors for gaze behavior.

Another line of research has focused on the predictive value of low-level image features such as contrast, color, edge density and, for dynamic scenes, motion. A range of algorithms exists to extract these features in images and videos and condense them into one *low-level saliency* value between 0 and 1 for each pixel, resulting in topographic low-level saliency maps^14^. Low-level saliency has been shown to explain fixation patterns for a variety of naturalistic and abstract images^15,16^, as well as naturalistic videos^12,17,18^ and has been argued to be a biologically plausible model of early visual processing^19^.

The influence of social stimuli and visual low-level saliency on eye movements have only recently been studied within the same datasets, and rarely in direct juxtaposition. During face perception, it was shown that facial regions diagnostic for emotional expressions received enhanced attention irrespective of their physical low-level saliency^20^. Birmingham and colleagues found social areas in an image to be a better predictor for fixation behavior than low-level saliency^21,22^. Other studies found faces to outperform low-level saliency on gaze prediction in dynamic scenes showing conversations between persons^7^ and documented higher predictive power for faces than for low-level saliency for adult participants watching a comic clip, although faces were not controlled for low-level saliency in this particular analysis^23^. Several studies reported an improvement of low-level saliency-based models by including faces as predictors^9,24,25^. Xu and colleagues included a variety of predictors at pixel level (color, intensity, orientation), object-level (e.g., size, solidity) and semantic level (e.g., face, gazed-at objects, text) and found higher weights for the combined predictors at the semantic level than at pixel- and object-level^26^.

Despite recent recommendations of increasing the ecological validity in social attention research^27^, several studies utilized impoverished stimuli such as schematic depictions of faces that are typically stripped of context or background information^20^. While this research strategy can illuminate basic attentional principles, its results may not easily extrapolate to real-world attentional phenomena, where faces are only one feature among many competing for an observer’s attention. Furthermore, most studies that do attempt to study social attention using contextually rich scenes typically do so using static images^4,9,26,28^. However, as motion is ubiquitously present in virtually all everyday situations and has been shown to be the strongest single predictor for gaze allocation^17,29^, video stimuli seem advantageous when investigating social attention compared to static stimuli. Moreover, it was demonstrated that participants show more consistent eye movement patterns when viewing videos compared to static images^30,31^, thus indicating a potentially higher predictive value of basic stimulus properties on visual exploration.

In order to address these issues, the current study followed the cognitive ethology approach mentioned earlier^27,32,33^. We used uncut, dynamic scenes showing naturalistic situations with no artistic ambition. By incorporating both low-level features such as motion and social information into one analysis, we aim at further illuminating the determinants of visual attention under ecologically more valid conditions. Importantly, gaze data was analyzed using a generalized linear mixed model (GLMM) approach. This allows for estimating several features’ unique contribution to fixation selection even in cases of co-variations between predictors. Specifically, this approach allows for crystallizing the effect of social information on gaze allocation even when, as it naturally occurs in real situations, depicted persons move or become visually salient in other respects. We hypothesized the performance of low-level saliency-based models to be poorer in social scenes compared to non-social scenes^22^. Furthermore, we expected social information to be a significant predictor for gaze behavior, even when controlling for low-level saliency and centrality.

A second rationale behind employing contextually rich video stimuli is linked to, but partly independent from the concept of ecological validity: by deliberately omitting standardization of stimuli on many dimensions, we intended to identify only robust attentional effects which are independent of idiosyncrasies in the experimental setup. Several intriguing experiments have demonstrated heightened sensitivity, but degraded reproducibility as a result of strict experimental standardization in animal research^34,35^, and the theoretical considerations employed to explain these findings^36^ seamlessly extend to human behavioral sciences. This idea is not entirely new to psychological experimentation, as documented by a general acceptance to leave plenty surrounding conditions unstandardized (e.g., time of the day, day of the week, participant’s mood and appetite, weather, room temperature, room smell, experimenter mood, air pressure), even though they may be expected to produce effects in some circumstances. The video stimuli used in the present experiment extended this rationale by varying on a large number of dimensions: general semantics of the scene, composition, brightness, lighting, color, amount of movements, type of movements, direction of movements, camera movement, appearance or disappearance of objects or persons, attractiveness of persons, to name just a few. To our opinion, adopting a cognitive ethology approach not only encompasses investigating social attention under naturalistic conditions, but also assessing whether social attentional mechanisms become tangible when considerable amounts of external variance are at play, as one would expect in naturalistic situations. In order to estimate the robustness of attentional effects, we will not only estimate the predictive value of social attention and low-level saliency throughout the entire dataset, but also examine their intra-individual consistency along the various video stimuli.

As an additional experimental manipulation, we compiled the stimulus material such that video clips differed in their affective quality. It is a well-established finding that threatening stimuli^37,38^, but also emotional stimuli in general^39,40^ attract attention and are processed preferentially. The majority of studies in this field employed static stimuli with drastic differences in valence like images selected from the International Affective Picture System (*IAPS*)^41^. By contrast, and again along the idea of a cognitive ethology approach, the current study aimed at investigating whether emotional quality affects gaze allocation when viewing naturalistic videos, in which differences in perceived valence are within the range of what persons typically encounter in their lives. Recordings of autonomic nervous system activity were additionally obtained to confirm the affective quality ratings. We hypothesized social features that contribute to the affective quality of the stimulus to gain weight in predicting gaze allocation at the expense of the influence of low-level visual features. To the best of our knowledge, social attention has not been studied before within such a setup of naturalistic affective videos whilst statistically controlling for low-level physical low-level saliency.

## Materials and Methods

### Participants

Thirty-two participants (*M* = 27.84 years, *SD* = 7.46 years, 7 males, 23 students) took part in this study. The sample size was determined a priori to detect a medium effect size of *d* = 0.50 in a one-tailed paired comparison with a power of at least 0.85. No participant reported a history of psychiatric or neurological illness or taking centrally-acting medication. All participants had normal or corrected-to normal vision. The study was approved by the ethics committee of the German Psychological Society (DGPs) and conducted in accordance with the Declaration of Helsinki.

### Stimuli

The participants viewed, in a randomized order, 90 complex naturalistic video clips of a duration of 20s each, depicting a variety of indoor (e.g., private homes, public buildings, public transport) and outdoor (e.g., streets, countryside, beach) scenes (for a description of some of these videos, see online supplement). Participants were not given any task or external motivation, but were instructed to freely view the scenes as though they were watching television. Sound was turned off in all videos. Forty-five of the video clips contained human faces and typically other body parts and were categorized as “social” (e.g., people walking in the streets or playing a ball game), while the remaining 45 clips did not show human beings (e.g., a train driving by, a scene in a forest). All videos were either obtained from publicly available online streaming services (e.g., www.youtube.com) or filmed by ourselves. We made sure not to use popular videos in order to reduce the risk of displaying a video to a participant who has viewed it before. No participant reported having seen any of the videos before when asked to disclose what had drawn their attention. Videos were required to depict situations that one could encounter in real life, as opposed to scenes that are primarily filmed for their artistic value. Moreover, we made sure that the persons appearing in the videos were unknown (i.e., no famous persons). Unlike impoverished stimuli sets often used, our video clips included a variety of visual information both in the back- and foreground, depicting a complex set of human actions and natural events. They were filmed with unpretentious camera movements and no cut. For the social as well as the non-social scenes, we made sure to include positive, negative and rather neutral clips. However, this a priori selection was only done to ensure sufficient variation in affective quality and the analyses were calculated using individual affective ratings of each participant. All clips had a resolution of 1280 × 720 pixels and a frame rate of 30 frames/s.

### Apparatus

Video clips were presented centrally on a 24-inch LCD monitor (LG 24MB65PY-B, physical display size of 516.9 × 323.1 mm, resolution of 1920 × 1200 pixels). Viewing distance amounted to approximately 50 cm, resulting in a visual angle for the videos of 38.03° horizontally × 21.94° vertically. Eye movement data were recorded from the right eye using an EyeLink 1000plus system (SR Research, Ontario, Canada) with a sampling rate of 250 Hz. Head location was fixed using a chin rest and a forehead bar.

Autonomic responses were continuously recorded at a sampling rate of 500 Hz during stimulation using a Biopac MP150 device (Biopac Systems, Inc.). Skin conductance was measured at the thenar and hypothenar eminences of the participant’s non-dominant hand by a constant voltage system (0.5 V) using a bipolar recording with two Hellige Ag/AgCl electrodes (1 cm diameter) filled with 0.05 M NaCl electrolyte. An electrocardiogram (ECG) was recorded using mediware Ag/AgCl electrodes (servoprax, Wesel, Germany) attached to the manubrium sterni and the left lower rib cage. The reference electrode was placed at the right lower rib cage. Stimulus presentation and data collection were controlled using the Psychophysics Toolbox^42^ on MATLAB R2011b (MathWorks, Natick, MA, USA), and the EyeLink Toolbox^43^.

### Procedure

Participants were invited to the laboratory individually and informed about the purpose of the study. Upon completing an informed consent form and a sociodemographic questionnaire, they were connected to the measurement instruments and given a detailed verbal explanation of the experiment. The 90 video clips were randomly sorted for each participant and presented in three blocks containing 30 clips each. Participants were asked to hold their heads still during the blocks, but allowed to sit comfortably or stand up between the blocks. The eye tracking system was calibrated and validated before each block using a 9-point calibration grid. Furthermore, a central fixation cross was presented for a randomly selected time interval between 5 and 9s before each video clip, and participants were asked to fixate it. The participants were given the instruction to watch and freely explore the video clips similar to watching a television program. Heart rate and skin conductance were recorded only during this part of the experiment.

Subsequently, participants watched the clips for a second time in the same order as before and rated them for arousal and valence using the Self-Assessment Manikin^44^ on a scale from 1 to 9. For the participants, about 45 minutes passed between watching a video a first and a second time. The Self-Assessment Manikin, which is routinely used in psychological research on emotional processing, involves a numerical scale which is accompanied by simplified drawings of a person in order to illustrate the concepts of valence and arousal with facial expressions and other comic-style visualization techniques. Additionally, we constructed a 9-point personal relevance scale by adopting non-verbal, graphic representations similar to those used for arousal and valence. Participants were asked to state, in a broad sense, to what degree each depicted scene had a personal relevance to them. To illustrate the abstract idea of relevance, the manikins were color coded using various shades of grey (darker colors = higher relevance). Finally, participants filled out several psychometric tests and questionnaires which are not part of this study.

### Data processing

Image processing was performed using MATLAB R2011b (The MathWorks, Natick, MA,USA). We computed low-level saliency maps for each video frame using the *GBVS* algorithm^15^. The channels “DKL Color”, “Intensity”, “Orientation” and “Flicker” were integrated into the maps with equal weights. In order to reduce the impact of strong changes in the low-level saliency distribution between successive video frames, we applied Gaussian blurring along the temporal dimension of the video data with a standard deviation of 2 frames. This technique aimed at better harmonizing the temporal reactivity of low-level saliency distributions with that of the human visual system, which cannot perform an entire action-perception cycle within the duration of one video frame. Each low-level saliency map was then normalized by dividing values for each pixel by the mean of the image, ensuring an average low-level saliency of 1 while preserving differences in low-level saliency variation between video frames.

Gaze raw data were analyzed using R for statistical computing (version 3.2; R Development Core Team, 2015). Gaze data during the first 150 ms after stimulus onset were excluded from the analysis to account for a minimum reaction time to leave the central fixation cross presented immediately before^45^. Data of each trial were corrected to account for drifts in head position. This was done using the mean valid gaze positions of the last 300 ms before stimulus onset as baseline. A recursive outlier removal algorithm was adopted to avoid correcting for drifts based on faulty gaze data (e.g., when participants did not fixate the fixation cross at some point during the last 300 ms of its appearance): Separately for x and y baseline coordinates, the lowest and highest values were both removed from the distribution, individually compared to the distribution of the remaining data and entered again if they were located within 3 standard deviations from the mean. This process was recursively applied to the remaining data until both the highest and the lowest data point met the criterion to be re-entered to be distribution. Subsequently, baseline position data from trials containing blinks or a discarded x or y component (*M* = 8.19% of all trials per participant, *SD* = 8.74%) were replaced by the mean of all valid trials, and baselines were subtracted from gaze data in each trial.

Since we preselected the videos with respect to their emotional valence, we primarily analyzed the influence of valence on attentional exploration and used arousal and relevance ratings to ensure comparability of video sets. Subjective valence ratings were expressed by the participants on a scale from 1 (very negative) to 9 (very positive). Intraclass correlation coefficients revealed varying interindividual consistency for valence (ICC = 0.65, 95% CI = [0.58, 0.72]), arousal (ICC = 0.47, 95% CI = [0.40, 0.56]) and relevance ratings (ICC = 0.19, 95% CI = [0.15, 0.26]). As a rule of thumb, coefficients between 0.60 and 0.75 are considered good, results between 0.40 and 0.59 are considered fair and results below 0.40 are considered low regarding interindividual consistency^46^. On the one hand, we directly used these ratings as a predictor in the GLMMs, on the other hand, we reclassified the videos into positive, neutral, and negative clips for additional analyses and manipulation checks. The thresholds between these three categories were adjusted individually for each participant to align the frequency with which each valence category was selected. For instance, if a participant tended to disregard the extremes of the rating scheme while showing a positivity bias, her ratings 6 and 7 may be relabeled as neutral (instead of 4 to 6 as one would define a priori). Specifically, an algorithm compared all possible permutations of the two thresholds and selected the combination that exhibited the smallest total difference in category size. As a result, *M* = 27.59 (*SD* = 7.12) videos were classified as negative, *M* = 32.09 (*SD* = 5.66) as neutral and *M* = 30.31 (*SD* = 5.16) as positive.

Autonomic responses were analyzed using the R software package as well. Skin conductance (or electrodermal activity, EDA) at trial start was subtracted from all data points within each trial, and data for each trial were averaged for further analyses. Heart rate (HR) data were calculated from the ECG recordings. First, R-waves were detected using a semi-automatic procedure. R-R-intervals were then converted to HR (in beats per minute) and a second-by-second sampling was applied^47^. The last second prior to stimulus onset served as prestimulus baseline and the corresponding HR value was subtracted from all values during stimulation (i.e., 20s). As for EDA, data were then averaged across each trial.

### Data availability

The datasets generated during and/or analyzed during the current study are available at https://osf.io/943qb/.

## Results

### Arousal, relevance and autonomic responses as function of presence of persons and valence

In order to confirm the expected modulation of autonomic responses by differences in perceived valence as well as the presence of persons, we first examined the influence of valence and presence of persons on arousal ratings and autonomic measures using 2 × 3 repeated measures ANOVAs with video category (social vs. non-social) and emotional valence ratings (individually reclassified as positive, neutral, and negative) as within-subject factors. In all statistical analyses, α was set to 0.05. For ANOVAs and regression models, η^2^_p_ and *R*^2^ are reported as effect size estimates, respectively. For all ANOVAs, degrees of freedom were adjusted using the Greenhouse-Geisser correction to account for possible violations in sphericity, and corresponding ε values are reported. Post-hoc pairwise comparisons were performed using Tukey’s HSD test.

**Figure 1:**
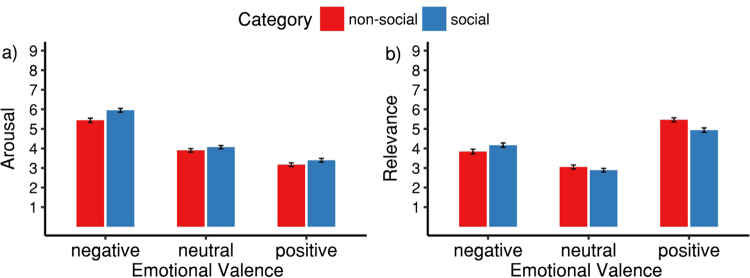
Effects of valence and presence of persons in videos on arousal and relevance ratings. Error bars indicate SEM.

Arousal ratings (Fig. 1a) were affected by valence (*F*(2, 62) = 62.66, *p* < 0.001, η^2^_p_ = 0.67, ε = 0.680) and presence of persons (*F*(1, 31) = 22.80, *p* < 0.001. η^2^_p_ = 0.42) but not by a valence × presence of persons interaction (*F*(2, 62) = 2.76, *p* = 0.085, η^2^_p_ = 0.08, ε = 0.788). Specifically, arousal ratings were higher for social compared to non-social videos, and higher for negative compared to neutral (*p* < 0.001) and neutral compared to positive videos (*p* = 0.002).

Relevance ratings (Fig. 1b) were affected by valence (*F*(2, 62) = 44.73, *p* < 0.001, η^2^_p_ = 0.59, ε = 0.870) and by a valence × presence of persons interaction (*F*(2, 62) = 9.68, *p* < 0.001, η^2^_p_ = 0.24, ε = 0.929), but not by presence of persons alone (*F*(1, 31) = 2.55, *p* = 0.120, η^2^_p_ = 0.08). Specifically, relevance ratings were higher for positive compared to negative (*p* < 0.001) and for negative compared to neutral videos (*p* < 0.001), resulting in a skewed U-shaped relation between valence and relevance. Relevance ratings for positive videos were furthermore higher than for negative videos (*p* < 0.001). For videos rated as positive, non-social videos were rated as more relevant (*p* = 0.001), whereas for videos rated as negative, social videos were rated as marginally more relevant (*p* = 0.071). There was no difference in relevance ratings between social and non-social videos rated as neutral (*p* = 0.376).

**Figure 2:**
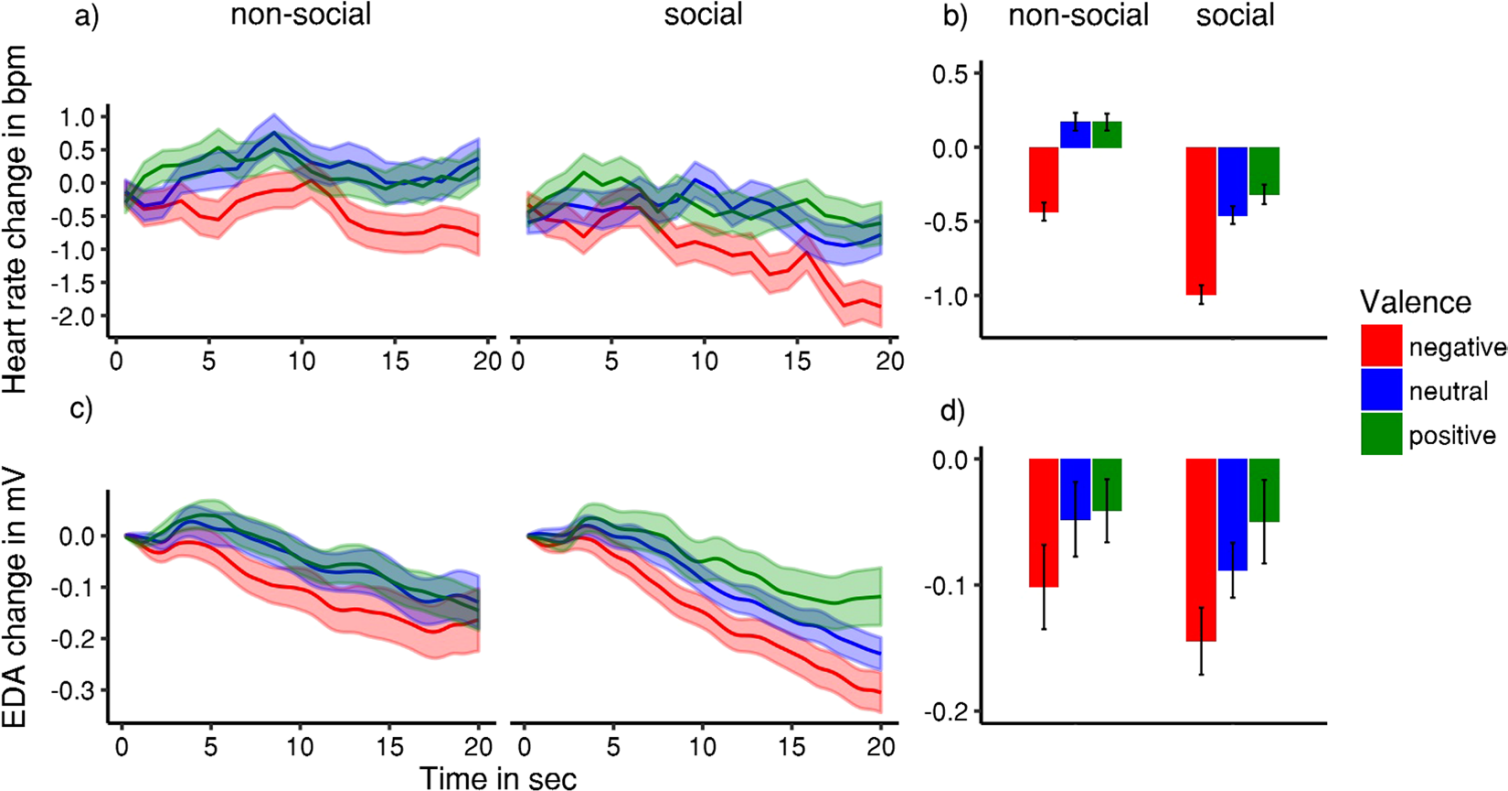
Physiological responses to different video categories. (**a**) Baseline-corrected heart rate change over time for non-social vs. social videos as a function of valence. (**b**) Heart rate change data aggregated across each trial. (**c**) Baseline-corrected change in electrodermal activity over time for social vs. non-social videos as a function of valence. (**d**) Electrodermal activity change aggregated across each trial. Ribbons and error bars indicate SEM.

Heart rate and skin conductance were measured as manipulation checks for differences in perceived valence (Fig. 2). We found a larger heart rate deceleration in social compared to non-social scenes (*F*(1, 31) = 7.47, *p* = 0.010, η^2^_p_ = 0.19) and an effect of video valence on heart rate changes (*F*(2, 62) = 4.12, *p* = 0.029, η^2^_p_ = 0.12, ε = 0.826), but no interaction of the two factors (*F*(2, 62) = 0.66, *p* = 0.521, η^2^_p_ = 0.02, ε = 0.929). Specifically, negative videos resulted in a stronger heart rate deceleration compared to positive videos (*p* = 0.020), while there was no statistically significant difference between negative and neutral (*p* = 0.109) or neutral and positive (*p* = 0.756) videos. Skin conductance was affected by valence (*F*(2, 62) = 3.81, *p* = 0.027, η^2^_p_ = 0.11, ε = 0.916), but not by presence of persons (*F*(1, 31) = 1.18, *p = *0.286, η^2^_p_ = 0.04) or an interaction (*F*(2, 62) = 0.26, *p = *0.774, η^2^_p_ = 0.01, ε = 0.979). Specifically, skin conductance was lower for negative than for positive videos (*p* = 0.021), but there was no statistically significant difference between negative and neutral (*p* = 0.312) or neutral and positive (*p* = 0.407) videos.

### Low-level saliency of looked-at pixels in different video categories

Next, we investigated the effect of valence and social content on the tendency to look at visually salient regions. To this end, we compared mean low-level saliency of all looked-at pixels by means of a 2 × 3 repeated measures ANOVA using video category (social vs. non-social) and emotional valence (positive, neutral, negative) as within-subject factors.

**Figure 3:**
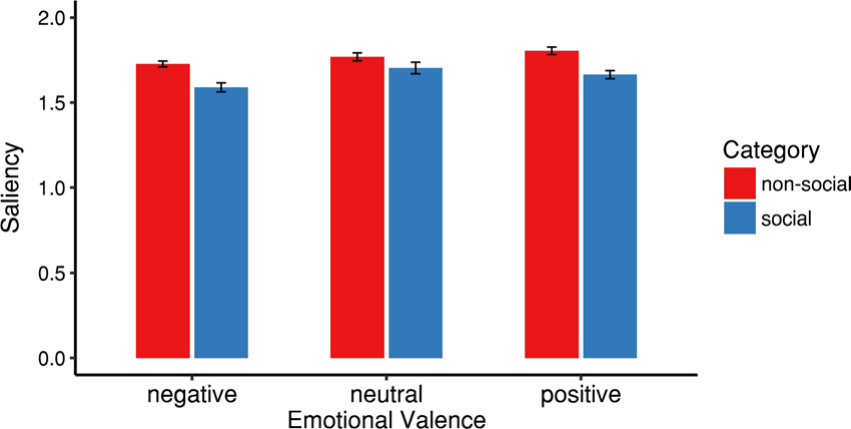
Mean low-level saliency of looked-at pixels in videos with and without presence of persons and for all three emotional valence subgroups. Error bars indicate SEM.

Low-level saliency of looked-at pixels (Fig. 3) was affected both by presence of persons (*F*(1, 31) = 93.29, *p* < 0.001, η^2^_p_ = 0.75) and valence (*F*(2, 62) = 7.01, *p* = 0.002, η^2^_p_ = 0.18, ε = 0.993). The interaction of both factors did not reach statistical significance (*F*(2, 62) = 1.61, *p* = 0.208, η^2^_p_ = 0.05, ε = 0.863). Specifically, low-level saliency of looked-at pixels was lower in videos with social information compared to videos without social information. Low-level saliency of looked-at pixels was also lower for negative than for neutral (*p* = 0.005) and positive (*p* = 0.006) videos, while there was no such difference between neutral and positive videos (*p* = 0.997). In all conditions, low-level saliency of looked-at pixels was higher than 1 – the value expected for a viewing behavior not guided by low-level saliency.

### Directly predicting gaze using GLMMs

While the analysis described above suggests a reduced influence of low-level saliency on visual exploration in the presence of social features, it cannot describe the relative contribution of both factors directly. Furthermore, it is susceptible to correlations between low-level saliency, social information and other potential predictors such as centrality. We therefore set up various generalized linear mixed models (GLMM) to directly describe the influence of centrality, low-level saliency, social information and valence on gaze behavior in the social videos.

This approach was adapted from Nuthmann and Einhäuser^48^. Social information in the videos was defined in a conservative manner, marking the human heads in each video frame with circular regions of interest (ROI). Analogous to the low-level saliency maps, these ROI maps consisted of ones representing pixels on heads and zeros representing pixels elsewhere. Centrality for each pixel was defined as inverse Euclidean distance to the center of the video. Predictor maps for each video frame were then divided into a 32 × 18 grid and data were collapsed within each of these 40 × 40 pixels grid cells. The size of the grid cells (2.5° × 2.5° of visual angle) approximated the functional field of the human fovea centralis. Values for low-level saliency, social ROI and centrality were then z-standardized to make resulting beta coefficients comparable.

In the GLMM, we implemented centrality, low-level saliency, social ROI as well as valence as fixed effects, low-level saliency × ROI as well as a low-level saliency × valence as interaction terms and participant ID and video ID as random effects. The response variable was binary and stated whether a given grid cell was fixated in a particular video frame or not. It was made accessible to linear modelling using the *probit* link function. In order to ascribe the same importance to both of the dependent variable’s states (looked-at vs. not looked-at) during modeling, we did not include all of the 575 grid cells per video frame which were not looked at. Instead, only one grid cell which was not looked at was randomly selected in addition to the looked-at grid cell. The resulting observation matrix consisted of approximately 1.73 million entries (32 participants × 45 videos × 600 frames × 2 grid cells). To compute regression weights for each predictor, we used the *glmer* function of the *lme4* package^49^ and the *bobyqa* optimizer. Since estimating goodness of fit is intricate for linear mixed models, we computed an analogue to the coefficient of determination, *R*^2^, but maintained this naming convention. This was accomplished by calculating the square of the correlation between observed data and data predicted by the model^50,51^.

We adopted an incremental model building strategy in order to assess the lower bound of each predictor’s contribution to explained variance. In the first model, we only included centrality as a fixed term, as this simple cue provides the most frugal gaze prediction. Second, we further included low-level saliency, a more complex but still bottom-up information channel. In a third model, the predictor social ROI, defined as depicted people’s heads, was included. In a fourth and fifth model, we further included a low-level saliency × ROI interaction term and a low-level saliency × valence interaction term.

Adopting an incremental model building strategy resulted in five models comprising an increasing number of predictors, with each model being nested in the consecutive one. This procedure allowed to estimate the lowest bound of a predictor’s contribution to explained variance, even with correlations among the predictors.

Since each model implemented random selections of only one out of 575 not looked-at-cells per video frame, we repeated the entire process 100 times and report averages and 95% confidence intervals of models’ characteristics in Table 1. Explained variance profited from the inclusion of low-level saliency and social ROI, which can be seen in the rising and non-overlapping 95% confidence intervals of explained variance in these models. Explained variance did not profit from further adding a low-level saliency × ROI interaction and a low-level saliency × valence interaction. The gain in explained variance when including social ROI in addition to centrality and low-level saliency was 6.62%, marking the most conservative amount of explained variance that can be attributed to social ROI alone. The three predictors centrality, low-level saliency and social ROI collectively explained 31.82% of the variance in gaze data.

**Table 1:** Results of hierarchical generalized linear mixed models (GLMMs) examining the contribution of different predictors for fixation selection.

		*Centrality*	Saliency	ROI	Saliency × ROI	Saliency × Valence	*R* ^*2*^
1	Centrality	0.554 [0.553, 0.555]					0.154 [0.153, 0.155]
2	+Saliency	0.266 [0.265, 0.268]	0.574 [0.572, 0.577]				0.252 [0.252, 0.253]
3	+ROI	0.288 [0.286, 0.289)	0.544 [0.542, 0.547]	0.506 [0.502, 0.509]			0.318 [0.317, 0.319]
4	+Saliency × ROI	0.287 [0.285–0.289)	0.526 [0.524–0.529)	0.509 [0.505, 0.512]	−0.103 [−0.107, −0.100]		0.318 [0.318, 0.319]
5	+Saliency × Valence	0.288 [0.286, 0.290]	0.528 [0.525, 0.530]	0.510 [0.507, 0.514]	−0.104 [−0.108, −0.100]	0.002 [−0.001, 0.004]	0.320 [0.319, 0.321]

Standardized regression weights and explained variance (R^2^) for models comprising an increasing number of predictors. Models are nested and include predictors in models shown above. All values were calculated by bootstrapping 100 sets of not-looked-at grid cells and performing GLMMs for each set. Estimates represent means of weights from each bootstrapping iteration. Values in brackets represent the 2.5^th^ and 97.5^th^ percentile rank as an unbiased estimate of the 95% confidence interval.

### Internal consistency of predictors

In this study, participants viewed a variety of video clips which varied on a number of dimensions. This stimulus set was thus very different from the well-standardized sets of stimuli that were used in many studies in the field, but aims at mapping the diversity and richness of every-day experiences. One may therefore object that models based on viewing behavior might not reflect general patterns in attentional allocation, but rather reflect idiosyncrasies of the individual video clips used. To our opinion, this concern can be refuted by demonstrating an intraindividual stability of viewing behavior across the different video clips. We therefore assessed the consistency of interindividual differences in viewing patterns across this diverse set of video stimuli. To this end, we computed generalized linear models as described above, but individually for each social video for each participant, each time describing the influence of centrality, low-level saliency and social information on gaze allocation (32 participants × 45 videos = 1440 GLMMs). The entire procedure was again repeated 100 times to account for influences of the random selection of not looked-at cells.

On average among the 100 bootstrapping draws, 87.1 out of 1440 models (6.05%, range: 68–100, 4.72–6.94%) could not converge. Beta weights in these models were replaced using a multiple imputations technique, Predictive Mean Matching^52^ (PMM). We created five imputed datasets for each iteration, resulting in a total number 500 datasets of predictor weights. Predictor weights were z-standardized along the video dimension to exclude effects due to general differences in the videos (e.g., flashing lighting, sudden appearance of fast objects or persons), but maintain the order and distances between predictor strengths for each participant. Resulting values were then tested for consistency across the whole set of videos using Cronbach’s α. Cronbach’s α is commonly used to quantify, on a scale from 0 to 1, the extent to which different items (e.g., from a questionnaire) are intraindividually consistent with each other, or, figuratively speaking, point into the same direction^53^. Internal consistency was α = 0.88 (95% CI = [0.87, 0.89]) for the predictor central bias, α = 0.75 (95% CI = [0.70, 0.79]) for the predictor low-level saliency and α = 0.87 (95% CI = [0.85, 0.89]) for social ROIs. These values indicate high intraindividual stability in the attentional preferences across the stimulus set. Interestingly, internal consistencies above 0.90 have been argued to indicate redundancy rather than consistency for personality questionnaires^54^. The currently observed values for a rich and ecologically valid set of videos can hardly be called redundant with regards to the video content and suggest high stability of attentional exploration patterns.

## Discussion

In the present study, we assessed how social information and affective quality of naturalistic video scenes affect gaze allocation in addition to low-level image features such as physical saliency and centrality. Low-level saliency and social information both had substantial and similarly large effects on gaze behavior. Additionally, participants exhibited consistent differences in their viewing behavior in terms of the predictive value of centrality, low-level saliency and social ROIs in the rich set of video stimuli used. This demonstrates that attentional mechanisms driven by centrality, low-level saliency and social information exert a similar influence across a wide range of situations and do not depend on subtle changes in the experimental setup. To our opinion, this finding provides backup for the assumption that comparisons of viewing behavior along different video categories (social vs. non-social, positive vs. neutral vs. negative) are informative and valid, even when standardization was reduced in the current study in favor of external validity.

Valence variation between videos, although arguably more subtle compared to standard image databases like the IAPS^41^, could be affirmed by a heart rate deceleration for negative and for social videos. These findings are in line with other studies that report heart rate deceleration in persons viewing negative compared to positive or neutral images, and a stronger heart rate deceleration for images containing human attacks compared to images containing animal attacks^55^. Variation in video valence and arousal was, however, not underpinned by lower skin conductance levels during viewing neutral as compared to negative or positive videos. It must be noted that, although autonomic measures are an established tool to quantify emotional reactions, findings differ on subgroups of emotions^56^. For instance, one study^57^ found an enhanced skin conductance response to threatening pictures, but not to pictures that were negative in other respects. We cannot rule out the possibility that the videos used in the present study elicited specific subgroups of emotions that we did not inquire in the questionnaires. Moreover, most studies on autonomic responses to affective stimulation used pictorial material^55,58^ and it is therefore unclear to what degree these findings translate to dynamic scenes such as the video clips used here. Finally, although arousal ratings were generally higher for negative video clips, no such increased arousal was evident for positive videos and overall arousal ratings were rather moderate. Interestingly, a U-shaped distribution was found for relevance ratings with emotionally charged video clips receiving higher ratings than neutral stimuli. Since participants viewed each video twice, modulations of perceived valence due to a mere exposure effect cannot be ruled out, although we expect such an effect, if present, to be subtle and not specific to individual videos^59^.

One line of gaze data analysis showed that participants looked at less salient areas in social as compared to non-social scenes, and at less salient areas in negative compared to positive and neutral scenes. This finding is in line with the concept of a default attention system that directs gaze towards visually salient objects, but is partly overridden by top-down processes such as the search for social or aversive information^24,60^. This pattern is comparable to arousal ratings where we also observed higher ratings for social than for non-social video clips but does not directly correspond to relevance ratings that showed an interaction between emotional valence and the presence of persons. However, the class of analysis used here does not allow for directly assessing the relationship between social information and gaze behavior, and is susceptible to correlations between social information and other information channels. Moreover, for the videos used in this study, arousal levels were not only higher for social videos, but also for negative scenes in general, thus potentially distorting comparisons between the different videos categories.

We therefore computed several generalized linear mixed models encompassing various cues to predict gaze behavior in social scenes. The best-performing model included centrality, low-level saliency and social information as predictors. Crucially, even though social information was defined conservatively as comprising only human heads, it yielded a regression weight nearly as large as low-level saliency and explained at least 6.62% of variance in gaze data in addition to centrality and low-level saliency. The negative low-level saliency × social information interaction may be interpreted as a ceiling effect in attentional allocation: when a scene area is both visually salient and exhibits social information, the resulting interest in this area is large, but smaller than would be expected if both attentional mechanisms were merely added, as assumed in a GLMM. However, it must be noted that the gain in explained variance due to a low-level saliency × social information interaction was not significant. A low-level saliency × affective quality interaction did not contribute to explained variance in this analysis. This finding may seem surprising considering that mean low-level saliency of looked-at pixels was lower in negative compared to neutral or positive videos. However, in the GLMM, variance can be allocated to the factors centrality as well as directly to the social regions of interest, possibly suppressing variance allocation for certain interactions found in other analyses. This finding highlights the complementary nature of the two gaze analyses we performed – comparing low-level saliency of looked-at pixels and directly predicting gaze location.

In the present study, low-level saliency was defined as a summation of feature maps in the GBVS algorithm^15^ with equal weights for each channel. Future studies may test the robustness of our approach by comparing models using several of the abundance of low-level saliency models that have been proposed^61^. Likewise, although summation of feature channels in low-level saliency algorithms is still widespread^9,61^, future research should test whether optimizing feature weights using one of several proposed approaches^62–64^ can even increase the amount of variance explained by low-level saliency. However, since simple summation of feature weights has been shown to outperform weight optimization techniques in several domains where large amounts of uncontrolled variance are at play^65,66^, simple feature weight summation appears to be a reasonable default strategy.

Operationalizing social information as only the heads of depicted persons may be seen not only as a conservative but even as an impoverished definition. For instance, two studies^4,22^ found not only heads to be fixated more often than other objects, but also – though less so – human bodies. A similar attentional bias was found for objects which are gazed at by depicted persons^25,26,67^. A comprehensive definition of social information would therefore need to include these and perhaps even more features. As incorporating more predictors into the model would increase the amount of variance explained, this further highlights that the importance of social information for fixation selection is still underestimated in the present study.

The material used in this study was informed by a cognitive ethology approach^33^. We avoided artificially impoverished stimuli such as images of faces shown in isolation, and instead presented participants a large variety of complex, dynamic and contextually rich video clips. By these means, we intended to elicit more natural and representative viewing behavior in our participants. The use of generalized linear mixed models allowed to guard the effect of social attention against possible confounds, thus serving as a counterpart for an experimental setup in which variables are not held constant between experimental conditions.

However, it should also be noted that the testing environment itself still significantly deviates from field conditions, since participants were asked to continuously attend to a video screen placed in front of them and were unable to interact with the persons and situations presented to them. Some authors^68^ argue that in real-world situations, fixation selection is often guided by an expectation to interact with an object. Furthermore, it was asserted that gaze behavior in real social situations is often guided by the knowledge that conspecifics may detect, and possibly reciprocate, one’s gaze^69^. Since these possibilities are disrupted in passive-viewing tasks with photos or videos presented on a computer screen, viewing behavior might systematically deviate from that found in everyday situations. One technical solution which has been argued to simultaneously excel at both ecological validity and experimental control is virtual reality^70^. With realistic forms of interaction implemented, this technology promises to close a gap between complex field studies and well-controlled laboratory experiments.

While this study demonstrated the relevance of social information for attracting gaze allocation, an open question is to what extent this form of attention must be seen as deliberate or automatic. Over the course of a 20-second-video, fixation selection can evidently not be entirely automatic. However, there are hints that saccades towards social stimuli may be reflexive in a time period right after the appearance of such stimuli. Several studies^20,28,45^ found saccades toward socially relevant regions so shortly after stimulus onset that they are not well explained by cortical routes of top-down information processing^71^. Instead, it has been proposed that faces or eyes are also processed in subcortical circuits involving the amygdala^72^ and might drive reflexive attentional capture via this route^73,74^. An interesting question arising in this context is whether naturalistic video clips contain identifiable key moments that elicit reflexive saccades towards social features.

A promising application of our paradigm may be the investigation of attentional mechanisms in patient groups. One clinical condition that typically entails altered face processing is social anxiety disorder. Patients with this disorder show an initial hypervigilance for social threat cues^75–77^, but avoid looking at the eyes region when presented with images for an extended period of time^78,79^. Patients with autism spectrum disorder were found to orient their gaze more towards salient areas and less towards faces, objects indicated by other persons’ gaze^80^ and eyes^81^ when viewing naturalistic images, as well as less towards faces and more towards letters when viewing dynamic scenes^82^, although findings were not entirely consistent^83^. With healthy observers, our GLMM-analysis of naturalistic videos yielded robust results while posing little cognitive demands to the participants. Together with the simplicity and naturalness of the task, this approach may be informative as well as feasible in a variety of patient groups for whom alterations in social attention are debated. Crucially, analyses based on GLMMs allow for detailed comparisons of model weights between individuals, stimulus material and their interactions^48,84^.

The present study gauged the importance of social information on gaze behavior when viewing naturalistic, contextually rich dynamic scenes, while at the same time controlling for the low-level information channels centrality and low-level saliency. With a conservative definition of social information, we found its influence on viewing behavior similarly large as low-level saliency. We furthermore argue that our research paradigm shows promise for investigations of social attention under a variety of circumstances, such as in clinical populations.

## Electronic supplementary material

Supplementary Information
