# Supplementary material for: Social content and emotional valence modulate gaze fixations in dynamic scenes
Source: Sci Rep. 2018 Feb 28;8:3804. doi: 10.1038/s41598-018-22127-w (PMC5830578; doi:10.1038/s41598-018-22127-w)
Supplement: Supplementary file 1 — Supplementary Information [file 41598_2018_22127_MOESM1_ESM.docx]

**Supplementary Information: Social content and emotional valence modulate gaze fixations in dynamic scenes**

Marius Rubo* & Matthias Gamer

Department of Psychology, Julius Maximilian University of Würzburg, Würzburg, Germany

***Description of stimuli***

In order to illustrate the variability of topics that were displayed in the current set of video stimuli, we provide a brief description of a set of 12 videos examples from the total pool of 90 video clips that were used in the present study. The emotional valence of these videos was classified by the experimenter when selecting the stimuli but we relied on individual ratings of emotional valence in all data analyses.

Social videos:

- Positive: Two men play with a soccer ball on a rural, spacious backyard surrounded by trees.
- Positive: A woman practices gymnastics involving a hula hoop on a beach in sunny weather.
- Neutral: A man pushes trolleys in an airport while other persons are waiting in a queue.
- Neutral: A man uses an electric screwdriver to work on a wooden rack in a workshop.
- Negative: Several men are involved in a brawl in a run-down urban environment.
- Negative: Grim-looking police officers discuss with disgruntled pedestrians in a run-down urban environment.

Non-social videos:

- Positive: Colorful balloons are being released into the air amidst several trees.
- Positive: The camera moves along a calm beach bordered by vegetated rocks.
- Neutral: A freight train drives along in a rural area.
- Neutral: Laundry hanged on a rope in a backyard is waving in the wind.
- Negative: The camera moves along a wasteyard full of shabby, rusted cars.
- Negative: The camera moves around an abandoned house with broken windows and surrounded by garbage and dry undergrowth.
